# Native Mitral Valve Endocarditis Complicated by Intermittent Heart Block With Spontaneous Recovery: A Case Report

**DOI:** 10.7759/cureus.94307

**Published:** 2025-10-10

**Authors:** Rayan Abou Zeid, Assaad Maalouf, Rachad Salame, Elie Stephan

**Affiliations:** 1 Department of Cardiology, Faculty of Medical Sciences, Lebanese University, Beirut, LBN; 2 Department of Cardiology, Faculty of Medicine, Saint George University of Beirut, Beirut, LBN; 3 Geriatric Unit, Department of Internal Medicine, Faculty of Medicine, Saint George University of Beirut, Beirut, LBN

**Keywords:** acute pulmonary edema, native mitral valve endocarditis, sinus rhythm recovery, surgical valve replacement, third degree atrioventricular block

## Abstract

Isolated native mitral valve endocarditis without aortic involvement is a rare but serious cause of complete heart block. In contrast, aortic valve endocarditis is a well-recognized cause of life-threatening conduction abnormalities due to its close anatomical relationship with the conduction system. We report the case of a 68-year-old female patient with Streptococcus viridans bacteremia, presenting with native mitral valve endocarditis complicated by complete heart block, with imaging and clinical findings confirming sparing of the aortic valve.

## Introduction

Infective endocarditis (IE) is a serious infection of the heart’s endocardial surface, often involving the valves and carrying significant risks, including life-threatening complications [1,2]. One such complication is complete atrioventricular block (complete heart block, CHB), a conduction system disturbance frequently linked to aortic valve endocarditis [3,4]. This association arises from the aortic valve’s anatomical proximity to critical conduction pathways, such as the atrioventricular node and the bundle of His. When infection spreads beyond the valve, forming a perivalvular abscess, it can disrupt these structures, leading to CHB [3,5]. Consequently, new conduction abnormalities in aortic IE often signal invasive disease, necessitating urgent imaging (e.g., transesophageal echocardiography) and potential surgery.

In stark contrast, CHB is exceptionally rare in isolated mitral valve endocarditis due to the mitral valve’s distance from the primary conduction system. A few cases have been reported in the literature, often associated with extensive perivalvular infection or abscess formation extending toward the aortic-mitral continuity or the membranous septum. In these reported cases, management usually required early surgical intervention in addition to targeted antibiotic therapy, particularly when the block persisted despite infection control [6,7]. Several mechanisms have been proposed to explain this rare phenomenon, including septic embolization to the conduction tissue, inflammatory edema compressing the atrioventricular node, or direct abscess extension along the fibrous cardiac skeleton [8]. Despite its rarity, CHB in mitral IE warrants attention, as it may reflect severe perivalvular involvement [6,9,10]. Clinicians should consider IE in febrile patients with unexplained heart block, even without aortic valve involvement, to ensure timely intervention.

This case explores the pathophysiology, diagnostic challenges, and management implications of this uncommon but critical association, emphasizing the need for heightened clinical awareness.

## Case presentation

We report the case of a 68-year-old female, an active smoker with a history of hypertension, who presented with a two-day history of chills, undocumented fever, and watery greenish diarrhea occurring twice daily, non-bloody and non-mucoid in nature. She also described a new erythematous papular rash predominantly affecting the upper flexural areas, neck, inframammary region, upper torso, and back, with mild linear accentuation in the skin folds. There was no mucosal involvement and no palpable lymphadenopathy. Her complaints were accompanied by right flank pain, while she denied respiratory symptoms, nausea, vomiting, urinary complaints, chest pain, dyspnea, palpitations, or syncope. One week prior to admission, she reported gingival trauma complicated by gingivitis and gum bleeding.

On admission, the patient was hypotensive, with systolic blood pressure ranging between 80-90 mmHg, and had a heart rate of 90 beats per minute on the 12-lead electrocardiogram. Respiratory examination was unremarkable, with clear bilateral breath sounds and no evidence of pulmonary congestion. Cardiovascular examination revealed no murmurs, rubs, gallops, or peripheral stigmata of infective endocarditis.

Laboratory investigations demonstrated elevated inflammatory markers, with increased C-reactive protein levels and neutrophilic predominance on the complete blood count, suggesting a systemic inflammatory or infectious process. High-sensitivity troponin was elevated, though serial measurements did not show dynamic changes, making acute coronary syndrome unlikely and supporting a diagnosis of non-ischemic myocardial injury, possibly related to sepsis-associated myocardial stress or inflammation. Serum electrolytes, renal function, and liver function tests were all within normal ranges. Urinalysis was negative for infection or hematuria, and cold agglutinin testing was negative (see Table 1).

**Table 1:** Laboratory Inverstigation on Admission and Follow-up ↑ denotes increased above the reference range. eGFR: estimated glomerular filtration rate

Prameters	Results	Reference Range
White blood cell count	9.5×10°/L	4.0-11.0×10°/L
Neutrophils	63%	40-70%
C-reactive protein (CRP)	32 mg/L ↑	< 5 mg/L
High-sensitivity troponin	115 → 125 → 124 ng/L ↑	< 14 ng/L
Sodium (Na^+^)	136 mmol/L	135-145 mmol/L
Potassium (K^+^)	3.8 mmol/L	3.5-5.0 mmol/L
Magnesium (Mg^2+^)	1.8 mg/dL	1.7-2.2 mg/dL
Creatinine	0.86 mg/dL	0.6-1.2 mg/dL
eGFR	69 mL/min/1.73m^2^	>60 mL/min/1.73m^2^
Liver fuction tests	Within normal limits	-
Urinalysis	Negative	-
Cold agglutinin	Negative	-

A contrast-enhanced abdominal CT revealed a well-defined hypodense lesion within the spleen, a finding consistent with splenic infarction. No radiological evidence of bowel ischemia or other acute intra-abdominal pathology was identified. Taken together, these findings supported a working diagnosis of enteritis complicated by splenic infarction, most likely attributable to an underlying thromboembolic or embolic process. Given the possibility of infective endocarditis with associated embolic phenomena, empiric treatment was initiated without delay. The therapeutic strategy consisted of systemic anticoagulation with low molecular weight heparin (LMWH) for presumed thromboembolic disease, combined with empiric intravenous antimicrobial therapy including ciprofloxacin and metronidazole to ensure broad coverage against gastrointestinal and anaerobic pathogens. In addition, intravenous fluids were administered to provide hemodynamic support and stabilize the patient’s overall condition.

Microbiological and cardiac findings

Two days later, blood cultures returned positive for Streptococcus viridans. Transthoracic echocardiography (TTE) revealed a suspicious mitral valve vegetation along with moderate mitral annular calcification, moderate mitral stenosis, mild mitral regurgitation, a mildly calcified aortic valve with mild aortic regurgitation, left atrial dilation, grade II diastolic dysfunction, and preserved left and right ventricular systolic function.

**Figure 1:**
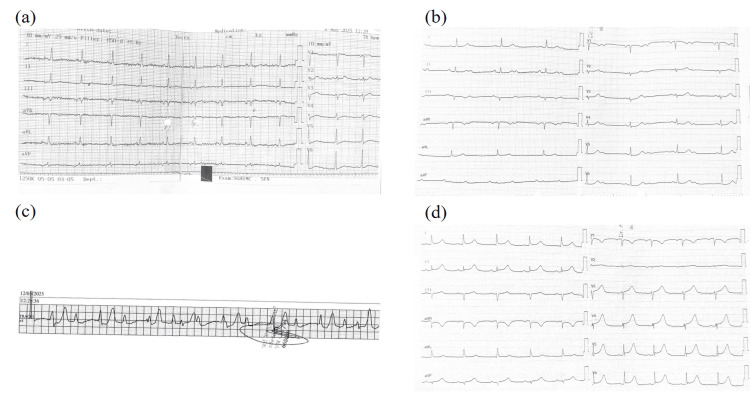
The figure shows (a) sinus rhythm with a ventricular rate of 78 bpm (b) a 2:1 atrioventricular (AV) block and (c) complete heart block with a ventricular rate of approximately 46 beats per minute (d) sinus rhythm with a ventricular rate of approximately 67 bpm

Shortly after, the patient developed progressive conduction abnormalities on ECG: evolving from sinus rhythm upon admission (see Figure 1A) to Mobitz type I four days after admission to 2:1 atrioventricular (AV) block during the night (see Figure 1B) then to complete heart block (see Figure 1C) after one day with a ventricular rate of 46 bpm. Despite this, the patient remained hemodynamically stable, and electrophysiology consultation deferred temporary pacing at that time. All beta-blockers were discontinued, and intravenous ceftriaxone was initiated. Continuous ECG telemetry was maintained during hospitalization, documenting the transition from complete heart block to partial AV block and eventual sinus rhythm recovery. This confirmed the transient nature of the conduction abnormality. A 24-hour Holter monitoring was planned after discharge to ensure long-term rhythm stability and exclude intermittent block recurrence.

Clinical course

From admission, the patient's electrolytes were within normal limits (Na⁺ 136 mmol/L, K⁺ 3.8 mmol/L, Mg²⁺ 1.8 mg/dL) and remained stable throughout, with no correction required. Serial evaluations ruled out potential metabolic or pharmacologic contributors to conduction block, including normal thyroid function, absence of ischemic changes on serial ECGs, and no evidence of myocardial ischemia on coronary angiography. No AV nodal blocking agents were administered during this period. Renal function was preserved (creatinine 0.86 mg/dL, estimated glomerular filtration rate (eGFR) 69 mL/min/1.73 m²), and there was no biochemical evidence of metabolic or electrolyte-related conduction disturbances.

Despite the absence of identifiable reversible causes, the patient developed a complete AV block, which persisted for two days. This subsequently transitioned to a 2:1 AV block, which lasted several more days before the patient spontaneously reverted to normal sinus rhythm on day 11 post-admission.

**Figure 2:**
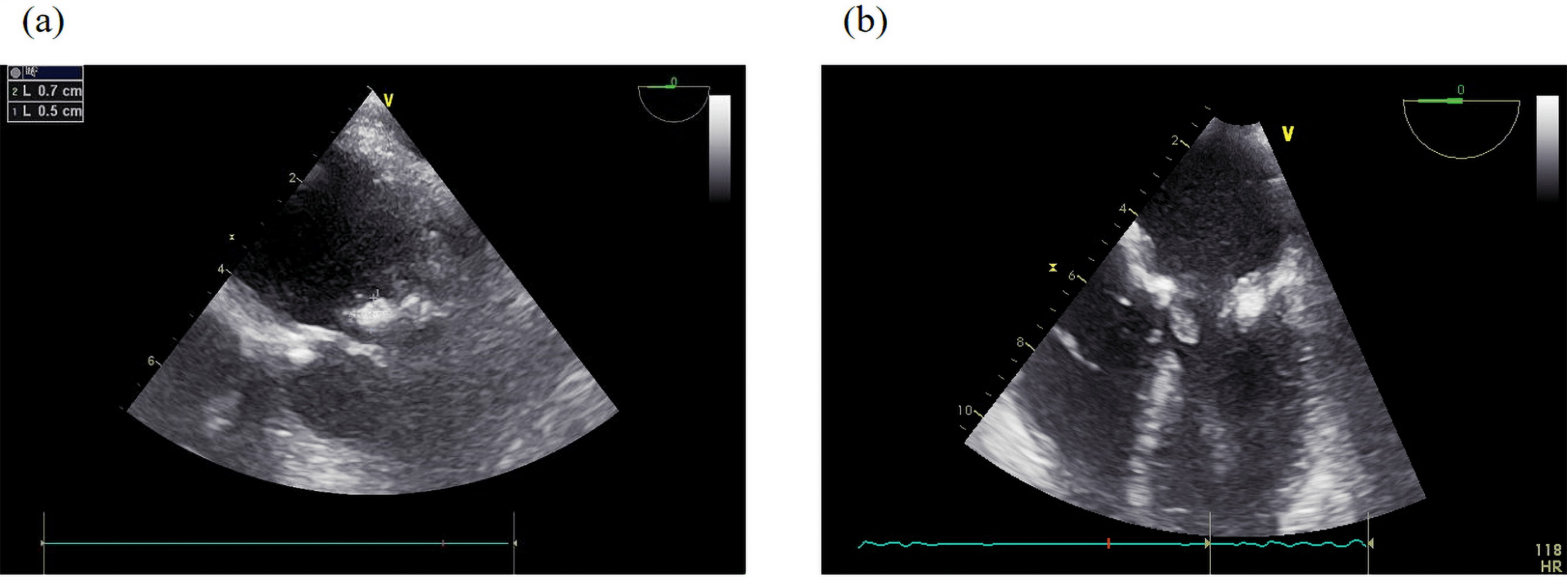
This figure illustrates (a) a focal thickening of 5–7 mm on the anterior leaflet and (b) a 7 mm thickening on the posterior leaflet, findings that are suggestive of vegetations.

A coronary angiogram performed on day seven demonstrated mild stenosis involving the proximal and mid segments of the right coronary artery, while only minimal non-obstructive disease was noted in the left anterior descending and left circumflex arteries. Around the same period, a transesophageal echocardiogram (TEE) revealed interval progression of mitral valve involvement. Specifically, a focal thickening of approximately 5-7 mm was observed on the anterior mitral leaflet, a finding suggestive of vegetation (Figure 2A), while a similar 7 mm thickening was detected on the posterior leaflet, likewise consistent with early vegetative changes (Figure 2B). In contrast, the aortic valve demonstrated only mild leaflet calcification without evidence of vegetations or clinically significant hemodynamic compromise.

Shortly thereafter, the patient experienced acute pulmonary edema, accompanied by worsening dyspnea and oxygen desaturation. Diuretic therapy was escalated, and supplemental oxygen was provided.

**Figure 3:**
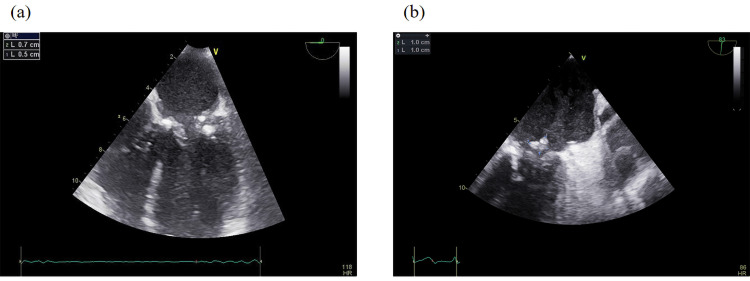
The figure shows (a) a 5 × 7 mm sessile mass on the ventricular aspect of the anterior mitral leaflet, and (b) a 10 × 10 mm mobile, fragmented vegetation on the posterior mitral leaflet protruding into the left atrium.

**Figure 4:**
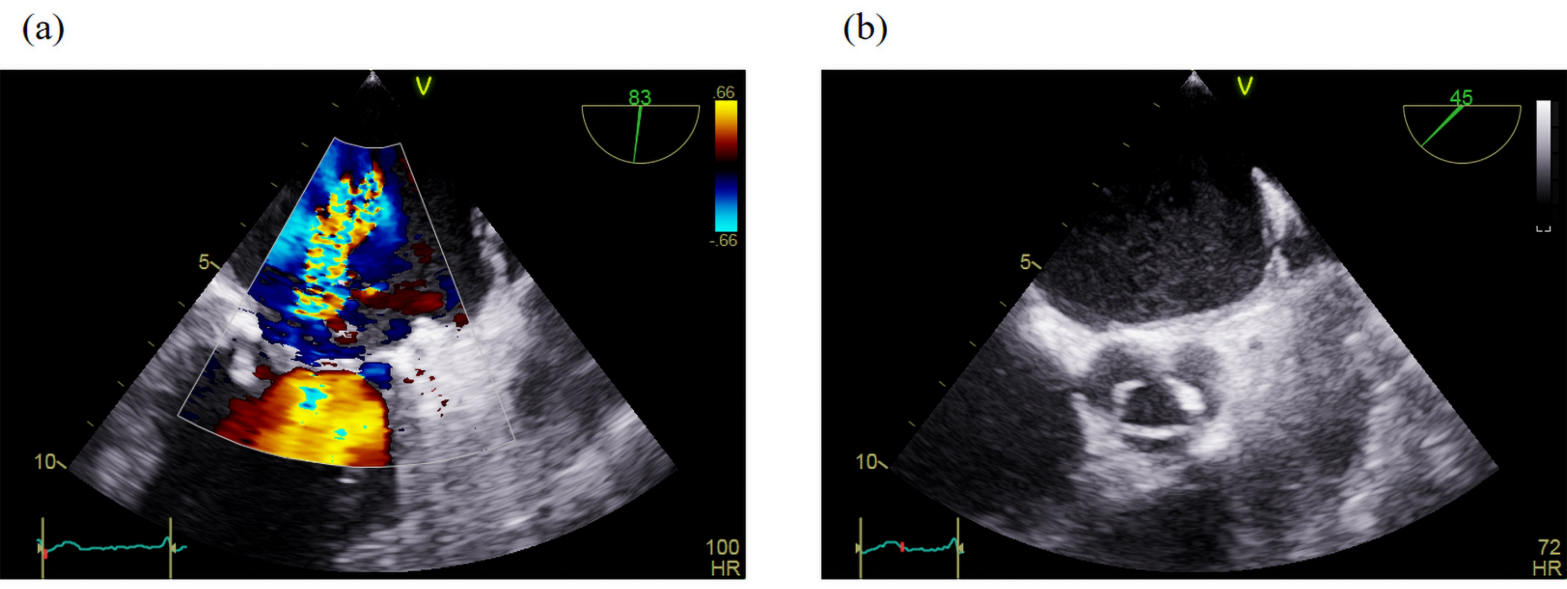
The figure illustrates (a) progression of mitral regurgitation from mild to severe, and (b) a mildly calcified aortic valve showing mild regurgitation without evidence of stenosis.

A repeat TEE, performed one week later, demonstrated marked progression of valvular pathology. On the anterior mitral leaflet, a sessile mass measuring approximately 5 × 7 mm was identified on the ventricular surface (Figure 3A). The posterior mitral leaflet exhibited a larger, mobile, and fragmented vegetation measuring 10 × 10 mm, prolapsing into the left atrium (Figure 3B). Mitral regurgitation had worsened substantially, progressing from mild to severe, with evidence of an eccentric jet and loss of leaflet coaptation (Figure 4A). In addition, the inferior vena cava was dilated with reduced respiratory variation, consistent with elevated right atrial pressures. The aortic valve remained mildly calcified and showed only mild regurgitation without stenosis (Figure 4B). Collectively, these findings indicated rapidly progressive infective endocarditis with severe mitral involvement, high embolic risk, and deteriorating hemodynamic status.

Management

Given the worsening valvular destruction and severe mitral regurgitation, the patient was started on continuous IV Lasix (250 mg) and referred urgently for surgical intervention. She underwent mitral valve replacement with a prosthetic valve. During this period, a CT pulmonary embolism (PE) protocol was performed and ruled out PE and deep vein thrombosis (DVT), prompting discontinuation of anticoagulation. The patient continued on intravenous antibiotics and was discharged home after clinical stabilization.

## Discussion

IE is a microbial infection of the endocardial surface of the heart, most frequently involving the cardiac valves, particularly in patients with underlying structural heart disease, prosthetic valves, or predisposing risk factors such as intravenous drug use or immunosuppression. Native valve endocarditis remains a serious clinical condition associated with high morbidity and potential for life-threatening complications [2].

Clinically, patients with IE may present with a broad spectrum of symptoms, including fever, chills, malaise, and the development of a new or changing murmur. Diagnosis is guided by the modified Duke criteria, with a major emphasis on positive blood cultures and evidence of endocardial involvement, typically evaluated by transthoracic or transesophageal echocardiography. Additional investigations such as ECG, inflammatory markers, and imaging studies help assess extent and complications [1].

Although conduction abnormalities such as AV block are well-documented complications in aortic valve endocarditis, they are rarely reported in cases of native mitral valve endocarditis. The development of complete heart block in this setting is highly unusual and typically indicates extension of the infection beyond the valve, such as perivalvular abscess formation or spread of inflammation toward the conduction system, particularly the AV node or bundle of His, which are anatomically adjacent to the aortic-mitral continuity [5,11].

Our patient presented with native mitral valve infective endocarditis, confirmed by positive blood cultures and echocardiographic evidence of vegetation on the mitral valve. During hospitalization, she developed a complete heart block, a rare complication in mitral valve endocarditis, raising concern for possible deep tissue invasion or abscess formation. However, serial echocardiography did not initially demonstrate a clear abscess. Notably, the patient spontaneously reverted to sinus rhythm without the need for temporary or permanent pacemaker insertion, suggesting transient involvement of the conduction system without irreversible structural damage.

The transient AV block in this patient may have resulted from inflammatory edema or localized ischemia affecting the conduction tissue near the aorto-mitral continuity, rather than fixed structural destruction. This mechanism aligns with reports suggesting that reversible inflammation can impair conduction during active infection and recover following antibiotic therapy. Other reversible causes of AV block, including electrolyte disturbances, myocardial ischemia, medication effects, and metabolic derangements, were systematically ruled out during hospitalization.

Furthermore, the patient’s age and mild aortic valve calcification may have contributed to a reduced conduction reserve, predisposing her to transient block under inflammatory stress. Although the aortic involvement was mild, shared fibrous continuity between the aortic and mitral annuli may explain limited conduction disturbance without extensive anatomical invasion.

Despite resolution of the heart block, the patient’s clinical course deteriorated. She developed worsening dyspnea and signs of pulmonary edema, prompting repeat TEE, which revealed a significant increase in vegetation size and the development of severe mitral regurgitation. This deterioration likely reflected progressive leaflet destruction and worsening valve dysfunction due to ongoing infection. In the context of hemodynamic instability and refractory symptoms, the decision was made to proceed with mitral valve replacement surgery.

A review of the literature identified only six previously reported cases of mitral valve endocarditis complicated by CHB, the majority of which were associated with large vegetations or the presence of periannular abscesses, particularly involving the mitral-aortic intervalvular fibrosa [4-7,9,10]. In nearly all cases, permanent pacemaker implantation was ultimately required due to either persistent AV block or irreversible structural damage to the conduction system.

In contrast, our case is notable for the spontaneous recovery of AV conduction prior to surgical intervention, with no requirement for pacing. The patient experienced transient CHB that progressed to 2:1 AV block, ultimately reverting to sinus rhythm by day 11 of hospitalization.

The mechanism of this spontaneous resolution is not fully understood, but several factors may have contributed. One possibility is that the conduction disturbance was caused by transient inflammation or infection in close proximity to the conduction system, which subsequently resolved with appropriate antibiotic therapy and time, allowing for functional recovery. Additionally, the absence of imaging evidence of a periannular abscess and lack of gross structural damage to the annulus or adjacent tissues may have preserved the integrity of the conduction pathway, facilitating reversible conduction delay rather than permanent block.

This case highlights several key clinical insights:

Complete heart block, although rare, can occur in native mitral valve endocarditis, and should prompt thorough evaluation for perivalvular extension, particularly abscess formation.

Spontaneous recovery of conduction is possible, especially in the absence of abscess or anatomical disruption on echocardiography.

The absence of conduction abnormalities should not delay surgical referral when there is progressive valvular destruction, severe regurgitation, hemodynamic deterioration, or increasing vegetation burden.

Serial imaging with TEE plays a critical role in monitoring disease progression and guiding the timing of surgical intervention.

## Conclusions

In conclusion, our case adds to the limited number of reported cases describing complete heart block as a rare but serious complication of native mitral valve endocarditis, particularly in the absence of aortic valve involvement. What makes this case unique is the transient nature of the conduction disturbance, with spontaneous return to sinus rhythm despite the initial complete AV block, and no evidence of a perivalvular abscess on imaging. This challenges the widely held notion that complete heart block in the setting of endocarditis always necessitates pacemaker insertion or indicates deep tissue invasion.

However, the patient’s course also highlights another important dimension: while the conduction abnormality resolved, the infection continued to evolve, leading to progressive enlargement of the mitral vegetation, severe mitral regurgitation, and acute pulmonary edema, ultimately requiring surgical valve replacement. This reinforces the idea that spontaneous resolution of conduction issues should not delay surgical evaluation when other complications emerge.

This case highlights the atypical presentation and evolving course of mitral valve endocarditis. It emphasizes the need to consider endocarditis in patients with general symptoms and new conduction abnormalities, even without classic signs. Early diagnosis and regular echocardiography are essential for detecting complications and guiding individualized care.
